# Fitness Determinants of Influenza A Viruses

**DOI:** 10.3390/v15091959

**Published:** 2023-09-20

**Authors:** Emily Fate Griffin, Stephen Mark Tompkins

**Affiliations:** 1 Center for Vaccines and Immunology, College of Veterinary Medicine, University of Georgia, Athens, GA 30602, USA; 2 Department of Infectious Diseases, College of Veterinary Medicine, University of Georgia, Athens, GA 30602, USA; 3 Emory-UGA Centers of Excellence for Influenza Research and Surveillance (CEIRS), Athens, GA 30602, USA; 4 Center for Influenza Disease and Emergence Response (CIDER), Athens, GA 30602, USA

**Keywords:** influenza A virus, virulence, genetic fitness, disease transmission, review

## Abstract

Influenza A (IAV) is a major human respiratory pathogen that causes illness, hospitalizations, and mortality annually worldwide. IAV is also a zoonotic pathogen with a multitude of hosts, allowing for interspecies transmission, reassortment events, and the emergence of novel pandemics, as was seen in 2009 with the emergence of a swine-origin H1N1 (pdmH1N1) virus into humans, causing the first influenza pandemic of the 21st century. While the 2009 pandemic was considered to have high morbidity and low mortality, studies have linked the pdmH1N1 virus and its gene segments to increased disease in humans and animal models. Genetic components of the pdmH1N1 virus currently circulate in the swine population, reassorting with endemic swine viruses that co-circulate and occasionally spillover into humans. This is evidenced by the regular detection of variant swine IAVs in humans associated with state fairs and other intersections of humans and swine. Defining genetic changes that support species adaptation, virulence, and cross-species transmission, as well as mutations that enhance or attenuate these features, will improve our understanding of influenza biology. It aids in surveillance and virus risk assessment and guides the establishment of counter measures for emerging viruses. Here, we review the current understanding of the determinants of specific IAV phenotypes, focusing on the fitness, transmission, and virulence determinants that have been identified in swine IAVs and/or in relation to the 2009 pdmH1N1 virus.

## 1. Introduction

Influenza A virus (IAV) is a major human pathogen that causes 3–5 million cases of severe illness and anywhere from 290 to 650 thousand deaths annually worldwide [1,2,3]. IAV is also zoonotic, permitting interspecies transmission and possible pandemics, as was seen in 2009 with the introduction of a novel H1N1 virus into the human population. This novel virus first emerged in pigs in Mexico before spreading to humans in North America and disseminating worldwide, becoming the first influenza pandemic of the 21st century [4,5,6,7].

Influenza A virus is an enveloped, negative-sense single-stranded, segmented RNA virus in the family *Orthomyxoviridae*. The RNA genome comprises eight segments that code for 10–11 proteins. Each segment codes for at least one structural protein and is numbered from largest to smallest: polymerase basic 2 (PB2), polymerase basic 1 (PB1), which codes for the PB1 and PB1-F2 proteins; polymerase acidic (PA), hemagglutinin (HA), nuclear protein (NP), neuraminidase (NA); matrix (M), which codes for both the M1 and M2 proteins; and non-structural (NS), which codes for both the NS1 and NS2 proteins, the latter being also referred to as the nuclear export protein (NEP) [2]. Influenza viruses can undergo two different forms of change: antigenic drift and shift. Antigenic drift is the gradual accumulation of mutations caused by an error-prone RNA-dependent RNA polymerase with no proof-reading mechanism. These point mutations can accumulate to allow the virus to escape existing antibodies and cause seasonal outbreaks. Antigenic shift occurs when two virions infect the same cell, enabling the mixing of gene segments and resulting in a new virus. This genetic reassortment can allow for a novel subtype to emerge with the potential to cause a pandemic [8]. Wild aquatic birds are the reservoir host of IAV. However, through adaptation and antigenic shift, some subtypes have acquired the ability to infect and transmit among a wide variety of hosts, including humans, pigs, horses, cats, dogs, and marine mammals [9]. One of these essential adaptations is the receptor-binding specificity of the HA protein. The first step in virus infection occurs when the HA protein on the surface of the virion binds sialic acids located on glycoproteins on the surface of the target cell. In the avian gastrointestinal and respiratory tracts, the majority of sialic acids are linked to the penultimate galactose via an α-2,3 linkage. In contrast, epithelial cells in the human upper respiratory tract contain glycoproteins with α-2,6 sialic acid linkages, although there are α-2,3-linked sialic acids in the lower respiratory tract. Most avian influenza viruses preferentially bind α-2,3-linked sialic acids, while human and other mammalian IAVs bind α-2,6 sialic acids [10,11,12]. Importantly, there are hosts such as swine that possess both α-2,3 and α-2,6 linked sialic acids throughout their respiratory tract and are consequently susceptible to both avian and mammalian influenza viruses [13]. This mixture of avian and mammalian IAV receptors has led to swine being considered an influenza mixing vessel as they can be infected with both avian and mammalian IAVs, providing the opportunity for gene segment reassortment. The reassortment that occurs in swine plays a central role in emerging human pandemics and the adaptation of avian influenza to mammals, as viruses are able to undergo host adaptation by acquiring host-optimized gene segments from circulating endemic viruses. Out of the 144 possible subtypes of influenza, only a few are prevalent and currently circulating in humans (H1N1 and H3N2) and pigs (H1N1, H1N2, and H3N2). However, it has recently been shown that a single amino acid change in the HA protein can alter receptor binding from an α-2,3 to an α-2,6 specificity [14]. Identification of genetic changes that can lead to species adaptation, virulence in hosts, and pandemic potential enhances our understanding of the biology of IAV, aids in surveillance and virus risk assessment, and guides the establishment of counter measures for emerging influenza viruses.

Influenza A virus was first isolated from swine in 1930 by Richard Shope. This H1N1 IAV remained the dominant strain circulating in North American swine for more than 60 years, and so it is referred to as the classical swine influenza virus (classical swine) [15,16]. In 1998, a human-origin H3N2 triple reassortment virus became endemic in swine, adding to the subtype diversity circulating in North American swine. The H3N2 virus contained the NP, M, and NS gene segments from the classical swine H1N1 virus, the PB1, HA, and NA gene segments from a human seasonal H3N2 virus, and the PB2 and PA gene segments of an avian influenza virus [17,18]. This combination of six internal gene segments, NP, M, NS, PB1, PB2, and PA, was referred to as the triple reassortment internal gene cassette (TRIG). It supported a variety of HA and NA combinations, greatly expanding the diversity of IAVs circulating in swine [19]. The classical H1N1 and recent H3N2 viruses continued to co-circulate and reassort, producing an H1N2 virus that became endemic. Moreover, human IAVs continued to spill back into swine populations, adding to the genetic diversity of HA and NA gene segments. While the surface glycoproteins continued to diversify, the TRIG cassette became the dominant gene constellation circulating in North American swine. It has been observed in most fully characterized swine influenza viruses since 2000 [17,20,21,22,23].

The pandemic H1N1 of 2009 (pdmH1N1) emerged from multiple reassortment events. The virus that caused the pandemic contained gene segments from three distinct swine influenza lineages: five of the six gene segments (PB2, PB1, PA, NP, and NS) from the triple-reassortment North American swine lineage, the HA from a classical H1N1 swine lineage, and the M and NA segments from a Eurasian avian-like H1N1 swine influenza lineage [24,25,26]. This virus rapidly spread across the globe, infecting an estimated 25% of the population but fortunately causing only limited mortality [27]. However, the virus also quickly crossed back into swine in multiple reverse zoonotic spillovers. While the full pdmH1N1 virus itself has not persisted in swine, several of its gene segments have been maintained in swine through reassortment and, in some cases, have become dominant in current circulating swine influenza viruses [28,29,30,31,32,33,34]. Specifically, the PA, NP, and M gene segments from the pdmH1N1 have gradually replaced the corresponding gene segments in the TRIG cassette [35,36]. While the 2009 pandemic was considered to have high morbidity and low mortality, studies have linked the pdmH1N1 virus and its gene segments to increased disease in humans and animal models [4,37,38,39,40]. While pdmH1N1 replaced the H1N1 viruses circulating in humans and is now effectively a seasonal human IAV, H1N1, H1N2, and H3N2 viruses continue to co-circulate and reassort in endemically infected swine and represent a public health threat. This is evidenced by the regular detection of variant swine influenza virus infections in humans associated with state fairs and other intersections of humans and pigs [41,42].

Understanding the genetic determinants affecting virus fitness, virulence, and transmission, as well as the mutations that may enhance or attenuate these features, is critical for the risk assessment of zoonotic influenza viruses. Mutations that attenuate IAVs may also be used to develop live attenuated influenza virus vaccines. Here, we discuss the determinants that have been mapped to specific phenotypes in influenza viruses. Mutations in avian influenza viruses that are important for virulence or transmission in mammals have been robustly reviewed by Lloren et al. [43]. Therefore, we will focus on the fitness, transmission, and virulence determinants identified in swine influenza A viruses and/or in relation to the 2009 pdmH1N1 virus. Table 1 summarizes the determinants and mutations to be discussed.

## 2. Fitness Determinants of IAV

### 2.1. The PB2 Gene

The PB2 protein forms one-third of the RNA-dependent RNA polymerase (RdRp), binds to the cap structure of cellular mRNAs, and recruits them for the initiation of viral replication, referred to as cap-snatching. The PB2 subunit of the RdRp complex is an important virulence and host determinant of influenza viruses.

#### 2.1.1. Role in pdm2009

As stated earlier, the pdm2009 virus is made up of gene segments from a variety of sources. The PB2 and PA gene segments originated from an avian virus that had previously undergone reassortment and became incorporated into a swine virus in 1998 [18,44]. The pdm2009 virus does not contain the 627K or 701N mutations in PB2 that have previously been shown to increase viral polymerase activity and contribute to increased viral replication and pathogenicity in mammals of certain subtypes (see below). In fact, two studies found that the E627K or D701N substitutions in the pdm2009 virus did not lead to enhanced virulence in mice or ferrets or enhanced transmission in ferrets [45,46]. This suggests that there are compensatory amino acid residues that are aiding in replication. By using mutated viruses with a swine influenza backbone, Liu et al. found that a combination of PB2 with residues 271A, 590S, and 591R in the genetic background of a classical H1N1 swine influenza A virus with the avian-origin PB2 is critical for the viral replication and virulence of swine viruses in vitro and in vivo. They postulate that this combination of residues in the PB2 gene is a novel strategy used by both the pdm2009 H1N1 and currently circulating triple-reassortment swine viruses for efficient replication and adaptation in mammals [47]. Residue 591 is located close to position 627 in the three-dimensional structure of PB2. Therefore, it is proposed that the arginine at 591 can compensate for the absence of lysine at 627 to still allow efficient viral replication [48]. In mid-2009, a swine-origin virus was detected with the PB2 mutation E667G. It was previously proposed that this substitution in the H5N1 virus was under positive selection and could possibly contribute to sustainable transmission to humans [49]. However, a study conducted by Herfst et al. found that, similar to the E671K and D701N mutations, E677G in PB2 also did not impact virulence or transmission in mice or ferrets [45]. Additional compensatory residues in the avian-origin PB2 gene, supporting viral replication at a lower temperature, were described by Hayashi and colleagues. They found that the presence of V661A and A683T/A684S mutations in the PB2 gene enhanced viral replication at 34 °C, likely contributing to viral growth in the upper respiratory tract and consequent transmission. The authors found that 661A and 683T are conserved among avian viruses in addition to swine and pH1N1 isolates. However, while 684S is common in humans and pdmH1N1 viruses, 684A is conserved in avian and swine isolates. The variation in amino acids between pdmH1N1 and swine isolates at position 684 suggests perhaps an alanine-to-saline mutation at PB2 684 played a role in the emergence of the pdm2009 virus [50].

Comparing PB2 sequences between human pdm2009 isolates and pdm2009 swine isolates that were lethal in mice, Zhao et al. discovered a consensus mutation: T588I. They determined that this mutation is conserved through pdm2009 viruses and was rare in previous human isolates before 2009. They found that this mutation increased polymerase activity and viral replication in human and porcine cells. It also contributed to increased pathogenicity in a mouse model and was shown to regulate the host antiviral innate immune response pathways in vitro and in vivo [51].

#### 2.1.2. Mutations Involved in Pathogenicity, Virulence, Replication, and Zoonotic Transmission

The most studied amino acid of the PB2 gene is K627, which has been implicated as an important determinant of the host range of influenza A viruses. A study published in 1993 found that out of the eight avian influenza viruses studied, all contained a glutamic acid at position 627, while the twelve human viruses used had a lysine at that position. They observed that this lysine residue at position 627 was conserved across H1N1, H2N2, and H3N2 viruses between 1933 and 1975 and among influenza B and C viruses. The authors demonstrated that in their original form, the avian viruses did not replicate well in mammalian cells; however, with a Glu to Lys mutation at 627, they were able to replicate efficiently. Mutation back to the original amino acid restored the original phenotype [52]. Interestingly, in 2005, there was an outbreak of H5N1 in birds in western China. Upon sequencing 33 isolated viruses from deceased birds, they found that all the isolates contained a motif of basic amino acids in the HA, which is characteristic of highly pathogenic avian flu, a lysine mutation at position 627 in the PB2 gene indicative of enhanced ability to replicate in mammalian cells; and a deletion of 20 amino acids in the NA gene, which is also associated with high virulence [53]. Along these lines, Shinya et al. found that an E627K substitution in PB2 converted a non-lethal H5N1 virus isolated from a human to a lethal virus in mice [54]. Similar results were found using an H3N2 swine influenza virus; the authors found that the mutation K627E attenuated the replication of the virus in mammalian cells and a mouse model [55]. This mutated position was also found to be critical for conferring airborne transmissibility of H5N1 in ferrets [56]. Research groups have also found that residue 627 on PB2 is a determinant of the natural cold sensitivity of avian-derived polymerase complexes and that this may contribute to their inability to grow efficiently in the human respiratory tract [52,57]. As far as the mechanism behind the mutations at position 627, a few research teams have offered some suggestions. Mehle et al. proposed that the E to K mutation at 627 facilitates an escape from an inhibitory factor that restricts the function of avian-derived polymerase genes in human cells without reducing their relative infectivity [58]. Another proposed mechanism is that residue 627 affects the interaction of PB2 and NP, that PB2 and NP poorly associate with each other in the presence of glutamic acid, and that the association is restored in the presence of lysine [59]. Recently, researchers have found an interaction between PB2 and ANP32A, a cellular nuclear protein involved in transcriptional regulation and mRNA export, among other roles. It is thought that avian influenza viruses adapted to utilize the avian ANP32A protein as a host factor to enhance polymerase activity. However, avian viruses cannot utilize mammalian ANP32A proteins without the acquisition of host-adapting mutations, including E627K [60].

In addition to position 627, Bussey et al. identified PB2 residue 271 as a key position for polymerase function and viral growth in mammalian cells. A mutation from the avian-conserved threonine to the human-conserved alanine enhanced polymerase activity and the growth of a recombinant virus in vitro and in vivo. The authors propose that, consequently, residue 271 plays a role in the mammalian adaptation of avian influenza A viruses. Although, unlike the E627K mutation, the T271A mutation was not able to cause lethal infection on its own, the double mutant (271A/627K) showed higher lung viral titers than the E627K mutant alone. The mechanism of action behind residue 271 is yet to be determined [61].

Another PB2 residue that has been implicated in the ability of a virus to cross the avian-mammalian species barrier is position 701. Mutation of aspartic acid to asparagine at position 701 is sufficient to enable replication and virulence of H5N1 in mice. The D701N substitution in the PB2 gene occurs naturally in H5N1 viruses present in ducks and, consequently, may facilitate the transmission of these viruses to mammals [62]. Steel et al. found that D701N can functionally replace E627K with respect to the adaptation of an avian virus to a mammalian host. They found that when PB2-627 was a glutamic residue, the D701N mutation improved viral growth in mammalian cells and enhanced aerosol transmission between guinea pigs using recombinant H3N2 and H5N1 viruses [63]. Gabriel et al. found that the D701N mutation allows for the increase in PB2 in the nucleus of mammalian cells, which suggests that the adaptation of the polymerase to the nuclear import machinery plays a role in interspecies transmission [61,64].

The residue PB2 271A has been maintained in the majority of swine isolates since the introduction of triple-reassortment viruses around 1998, and all pandemic swine-origin influenza viruses have contained PB2 residues 271A/627E/701D [61].

An additional mutation was discovered in H5N1 human isolates from Indonesia. Indonesia, Vietnam, and Egypt have reported the highest number of human cases of H5N1 infection to date. These isolates had a PB2 gene containing a 526R residue but not the 627K residue common in H5N1 human isolate cases. Song et al. found that the mutation of PB2-526R to 526K abolished polymerase activity in a human isolate of H5N1 and that polymerase complexes containing PB2-526R derived from H7N9, H5N1, or H3N2 exhibited increased polymerase activity. They found that, in concert with mutation 627K, 526R caused enhanced replication and greater morbidity and mortality in mice. They propose that K526R is a new adaptation marker for H7N9 and H5N1 of Indonesian origin in humans and provides an extra advantage for H3N2 viral replication [65].

Serial passage of a pdm2009 virus strain through mice revealed a new mutation proposed to be a pathogenic determinant. The PB2-E158G mutation increased morbidity and mortality in mice and was shown to impart increased RdRp activity in human cells [66].

In a study seeking to identify novel molecular determinants of virulence in H5N1 viruses in mammals, three residues in the PB2 gene were found to enhance replication and virulence in an H5N1 isolate (PB2-I147T, K339T, and A558T). These mutations are found in nature, and H5N1 viruses containing these three residues have been circulating in birds for years. Nevertheless, these three residues alone were found to increase replication in mammalian cells. However, combined with PB2-627K, pathogenicity was further increased. This combination of mutations is not unprecedented; an H5N1 virus with these amino acids was isolated from a patient in China in 2009 [67]. A high percentage of swine viruses contain 147T and 588T residues, consequently passing these mutations to the pdm2009 H1N1 virus [68].

### 2.2. The PB1 Gene

The PB1 protein is the second component of the RdRp of the influenza virus. The polymerase is involved in both the transcription and translation of the virus genome. PB1 contains several functional domains; the N terminus binds to PA, and the C terminus binds to PB2; it also contains two regions that bind to vRNA [69].

#### 2.2.1. Role in pdm2009

The PB1 gene segment of the pdm2009 H1N1 virus originated from a human seasonal influenza H3N2, which acquired the gene from an avian virus in 1968 [70]. By using a computational algorithm on database-acquired genomes, Chen et al. demonstrated that 96.6% of avian viruses contained a serine at position PB1-216, and 98.8% of them contained a valine at position PB1-336. However, all the pandemic sequences contained a glycine at PB1-216 and an isoleucine at PB1-336. Therefore, positions 216 and 336 were considered to be host species-associated in H1N1 viruses; PB1-216G is the human-associated residue; and PB1-336I is associated with infections in both humans and swine [44,71]. Lin et al. investigated the biological significance of the PB1-S216G point mutation in human H1N1 viruses. They found that switching amino acids back and forth on the same PB1 backbone greatly affected virulence in mice, demonstrating the importance of PB1-216 as a virulence determinant for H1N1 in mice. A glycine at position 216 attenuated PR8, and by replacing glycine with the original serine, virulence was restored in the mouse model. They also found that switching from serine to glycine increased the mutation frequency of pdm2009 by reducing the fidelity of RdRp. Consequently, the authors found that the naturally occurring mutation at PB1 position 216 affected viral replication fidelity, virulence, and adaptability [44]. PB1 residues 473V and 598P have been shown to contribute to the polymerase activity of H5N1 viruses in mammalian cells. Residue 473V is conserved in the pdm2009 strain, and substitution of the valine by leucine led to decreased viral polymerase activity and a lower growth rate in mammalian cells, suggesting that the 473V residue plays a role in maintaining efficient virus replication of the pdm2009 virus [72].

The pdm2009 H1N1 virus does not produce a PB1-F2 protein; it is thought that the absence of this virulence factor, among others, is responsible for the low virulence that has been associated with pdm2009. The pdm2009 virus encodes a truncated 11-amino acid form of PB1-F2, which contains three stop codons preventing the full-length expression of the protein [73].

#### 2.2.2. Mutations Involved in Pathogenicity, Virulence, Replication and Zoonotic Transmission

A study by Chen et al. found that the incorporation of an avian PB1 gene into the background of human virus internal genes (here referring to PB2, PB1, PA, NP, M, and NS) significantly increased virulence in mice. They propose that the acquisition of an avian PB1 gene, which was seen in the 1957 and 1968 pandemic strains, may have been a critical factor in the early stages of the pandemic. This acquisition might have allowed the emerging reassortant virus to overcome competition with seasonal influenza viruses through enhanced replication or virulence [74]. Gabriel et al. found that the point mutations L13P and S678N in the PB1 gene, originally found in a lethal mouse-adapted H7N7 virus, individually caused enhanced polymerase activity. The 13P mutation is located within the PA-binding site (residues 1–25), and 678N is localized within the PB2 binding site (600–757) [75]. The authors propose that these mutations could improve the interplay of these subunits between each other and NP in a new host environment [76]. The amino acid 622 is located within one of the vRNA binding domains. A study in 2016 conducted by Feng et al. determined that a G622D mutation in the PB1 gene resulted in a 10% reduction in the protein to bind vRNA in vitro, a 40-fold decrease in polymerase activity, and a 500-fold attenuation of the virulence of H5N1 in mice [69]. The authors suggest that this attenuating mutation could be used in the development of future live attenuated vaccines against influenza viruses.

Kamiki et al. discovered a novel PB1 mutation (K577E) in H9N2 viruses that were serially passaged through mice. They found that this mutation increased pathogenicity in the mouse model and proposed that it could be one of the signatures for mammalian adaptation to avian viruses. They also found that the PB1-K577E mutation enhanced viral polymerase activity in human cells at both 37° and 33 °C, suggesting that this mutation may allow the virus to efficiently grow in the upper respiratory tract of humans, which is an obligatory phenotype of human-adapted viruses. This mutation has not been seen to occur naturally in H9N2 viruses and occurs very rarely in other subtypes. However, these findings aid in substantiating the role of the polymerase complex in the adaptation of avian viruses to a mammalian host [77]. In addition to the pathogenic findings from K577E, Katz et al. found that the M317I mutation in the PB1 gene correlated to the high pathogenicity of H5N1 viruses in mice [78].

Taubenberger et al. compared the 1918 H1N1, 1957 H2N2, and 1968 H3N2 pandemic human viruses, which each possessed a uniquely derived avian-like PB1 gene segment, aligning these gene segments to identify any parallel changes that could be attributed to human adaptation. They found that these three viruses differ from an avian consensus by 4–7 residues each, but only one change was shared among all three, PB1-N375S. The serine residue at 375 can also be found in swine and equine isolates. They found that, with a few exceptions, all human influenza proteins have a serine at this residue. Consequently, this residue could be a marker of avian-to-human adaptation and, as a result, should be included in surveillance panels [79].

#### 2.2.3. Enhancement of the Effects of Other Gene Segments/Co-Mutations

While PB1 mutations have specifically been seen to impact virulence, replication, and adaptability, they are also one subunit of the RdRp. It interacts closely with PB2 and PA, and logically, co-mutations have arisen. A study by Uraki et al. sought to determine the mechanism of pathogenicity of the pdm2009 virus. They found that lysine or isoleucine at position 340 or 649 of PB2 and threonine at position 667 on PB1 contribute to higher virulence in mice of a recombinant virus where the parental strain was not lethal. However, the authors did not see a difference in replication efficiencies between the virus containing these mutations and the parent strain. They note that positions 667 on PB1 and 649 on PB2 are in the interaction site between PB1 and PB2, and position 340 on PB2 is located in the cap-binding domain. No viruses possessing only one of these mutations have been found to occur naturally, indicating that the mutations may be co-dependent and provide an additional advantage in terms of stability or survival for viruses versus the presence of each mutation alone [75,80]. Wei et al. also sought to examine the effect of mutations on the pathogenicity of pdm2009. The authors created a recombinant virus with the same gene constellation as pdm2009 and passaged it through pigs. After nine serial passages, they obtained viruses with enhanced virulence and transmissibility. The authors found that a mutation that occurred in PB1 (A469T) conferred enhanced polymerase activity to the parental recombinant H1N1 RNP complex and allowed for contact transmissibility in guinea pigs. Through sequence analysis, this mutation has been observed to be conserved in pdm2009 isolates and, consequently, may be an important pathogenicity determinant of the pdm2009 strain [81].

PB1-F2 is a protein derived from the PB1 gene via an alternate reading frame at nucleotide position 120. PB1-F2 has previously been shown to contribute to viral pathogenesis in mice, causing weight loss, slower viral clearance, and increased lung viral titers [82]. It has been proposed that, through in vitro effects on monocytes, the PB1-F2 protein causes apoptosis of immune cells, leading to decreased antigen production and a consequent decrease in the adaptive immune response [83]. Using a reverse genetics system, Conenello et al. were able to demonstrate that PB1-F2 contributes to the virulence phenotype seen in highly pathogenic viruses. They found that the N66S mutation found in the 1918 H1N1 virus was a crucial factor in this phenotype. The authors were able to further correlate the N66S mutation to high pathogenicity across other viruses by aligning highly pathogenic viruses against low pathogenic viruses. They propose that this mutation, located in the mitochondrial targeting sequence, affects the proapoptotic function of the PB1-F2 protein, potentially increasing the induction of apoptosis [84].

### 2.3. The PA Gene

The PA protein forms the third component of the RdRp and contains an endonuclease active site, which plays a role in cap-snatching during viral mRNA transcription. In addition to the PA protein, the PA gene segment also encodes for PA-X. PA-X is a fusion protein incorporating the N-terminal of the PA protein with a short C-terminal domain encoded by an open reading frame (ORF) ‘X’ that is accessed by frameshifting [85]. PA-X can act as an mRNase and has been shown to have lineage differences in X lengths. While about 75 percent of isolated sequences have a 61-codon X-ORF and represent almost all host species and HA/NA subtypes, 25 percent of isolates have a 41-codon X-ORF. These shortened X-ORF viruses are predominantly from the pdm2009 H1N1 virus, and a subset are from swine H3N2 and H1N2 viruses. Jagger et al. used a strain of the 1918 H1N1 virus in a mouse model to support the hypothesis that the PA-X protein plays a role in modulating host gene expression during infection. They found that while PA-X was not required for viral replication, its absence resulted in greater clinical disease and inflammation in vivo [85].

#### 2.3.1. Role in pdm2009

The PA gene segment of the pdm2009 virus accompanied the PB2 segment from the avian virus that had previously recombined to create novel swine viruses in 1998 [18,44]. The pdm2009 virus possesses a stop codon in the X-ORF, which leads to a truncated protein [85,86]. Several studies have found that viruses with a full-length PA-X protein led to greater replication in A549 cells and greater replication and pathogenicity in mice in comparison to viruses with a truncated PA-X protein [87,88]. This is perhaps in part due to the ability of PA-X to counteract host anti-viral responses by degrading cellular genes associated with protein metabolism and repair. PA-X has been found to interact with NS1, a viral protein that can more specifically target and degrade host mRNA related to interferon signaling and cytokine release. The specific interaction between PA-X and NS1 is not observed in pdm2009 viruses as the NS1 protein is inactive, but enhanced activity of the PA-X protein has been observed [85,87,89,90,91]. The PA subunit of the polymerase complex is associated with binding to the cRNA promoter and, to a lesser extent, the vRNA promoter on the viral genome for replication. This occurs on the N terminus of the PA protein, which overlaps with the PA-X frame-shift site [92]. The N terminus has also been implicated in endonuclease and protease activities [93,94]. Bussey et al. found that in the case of the pdm2009 virus, the PA protein has a greater impact on polymerase activity than the PB2 protein in mammalian cells. They found that amino acids T85I, G186S, and L336M in a pdm2009 viral PA contributed to enhanced polymerase activity of an avian polymerase complex in mammalian cells. When these residues, found in the pdm2009 PA, were introduced into an avian virus, PA viral protein synthesis was enhanced, resulting in increased viral titers during replication. Further, residue 336M increased morbidity in mice. However, mortality was unchanged for all mutations [95]. Regarding mechanisms of enhanced polymerase activity, Lutz et al. found that T85I, G186S, and V100I were involved in the regulation of translation and the accumulation of viral mRNA. Specifically, through interactions with a host RNA-binding protein, GRSF1 [96]. Xu et al. sought to understand the potential consequences of viral genetic variations on the infection characteristics of pdm2009; they examined the genomic polymorphisms that occurred among 10 strains of the pdm2009 H1N1 viruses. They found five unique non-synonymous mutations present in a strain with greater replication ability and virulence in mice, which they believe to be critical molecular determinants for replication, virulence, and pathogenicity. The following amino acid substitutions may be determinants: PA A343T, PB1 K353R and T566A, and PB2 T471M. However, the authors were not certain if increased virulence was caused by just a single substitution or a combination of any of the above four residues [97]. Chen et al. conducted a large-scale scanning of influenza protein sequences to determine the adaptive mutations that contributed to the emergence of pdm2009 and how they can replicate and transmit in humans. They used entropy-based computation to determine eight signature positions specific to host species. Seven of these positions changed from avian to human-like signatures in recent ancestral swine viruses of pdm2009. However, the PA 356 residue retained lysine, an avian signature. The authors proposed that mutation of this residue from lysine to the human-like arginine present in pdm2009 viruses allowed the virus to replicate and transmit in humans [71].

#### 2.3.2. Mutations Involved in Pathogenicity, Virulence, Replication, and Zoonotic Transmission

By serially passaging an avian virus through mice, Song et al. discovered that the PA gene was a strong determinant of the virulence and replication observed in a lethal strain of virus resulting from passaging. Specifically, using reverse genetics, they were able to identify that PA T97I was essential for this virus’s virulent phenotype but did not significantly increase virulence when introduced into the PA of a less virulent virus. The authors proposed that the T97I mutation likely resulted from adaptation to a mammalian host and could be a determinant of efficient replication and virulence in mice [98]. Yamaji et al. found five amino acid substitutions in PA that increased the ability of an H5N1 virus to replicate in human cells and increased pathogenicity in mice (PA substitutions V44I, V127A, C241Y, A343T, and I573V). The authors suggested that these substitutions contributed to the adaptation of H5N1 to mammalian hosts [99].

Recently, Meng et al. defined four mutations in the PA gene of a Eurasian avian-like H1N1 swine influneza reassortant virus that increased replication and pathogenicity in mice, as well as increasing disease and transmission in ferrets. This novel reassortant virus A/swine/Liaoning/265/2017 (H1N1), shared the same gene constellation as an earlier isolate, A/swine/Guizhou/828/2016 (H1N1), but reverse genetics analysis showed the increased pathogenicity and replication mapped to the PA gene segment of the 2017 virus. Furhter analysis demonstrated that four mutations in the PA gene, V100I, N321K, I330V, and A639T, were responsibe for the increase phenotype. Specifically, V100I increases PA endonucleasue activity, while N321K together with I330V enhanced PA vRNA-binding. While a specific phenotype was not associated with the A639T mutation, the combination of the four amino acid changes conferred the increased replication and pathogenicity in mice, as well as the increased disease severity and aerosol transmission in ferrets [100].

#### 2.3.3. Enhancement of the Effects of Other Gene Segments/Co-Mutations

Kim et al. demonstrated that a K142Q mutation in the PA protein resulted in enhanced replication and pathogenesis in mice when in combination with PB2-627K in an H5N1 virus. They proposed that these results suggest a role for position 142 of PA in the adaptation and pathogenicity of H5N1 viruses in a mammalian host. The authors suggested that the mechanism of these results could occur via conformational changes in the N-terminal site of the PA subunit, potentially changing promoter binding [101]. Ilyushina et al. sought the molecular mechanism used by pdm2009 to effectively adapt to humans and explored the molecular determinants of host range and pathogenicity of pdm2009 viruses in mammals. They found that the combination of the PB2 mutations E158G/A and PA L295P was sufficient to enhance the transcription and replication activity of polymerase complexes of pdm2009 H1N1 influenza viruses in mice [102].

### 2.4. The HA Gene Segment

The HA glycoprotein on the exterior of the virion is responsible for recognizing and binding to sialic acids to initiate infection and acts as an antigenic determinant. Following endocytosis and a decline in pH in the endosome, the HA protein undergoes a conformational change. This exposes a fusion peptide, triggering endosome–virus membrane fusion and allowing for the release of the genome segments into the host cell cytoplasm [103].

#### 2.4.1. Role in pdm2009

As explained above, virulence determinants for influenza A viruses have been well documented. However, most of these virulence factors are absent in the early pdm2009 viruses. Consequently, Chutinimitkul et al. found it prudent to map factors that could reasonably evolve in pdm2009 isolates that would cause them to become more pathogenic. The authors found that the mutation D225G in the HA protein, while not impacting pathogenesis in mice, did alter receptor binding. They observed that this mutation enabled enhanced attachment of the virus to macrophages and type II pneumocytes in the alveoli. This phenotype has also been seen in fatal H5N1 and H7N7 infections [104,105]. They noted an increased affinity for α2,3-SAs while maintaining specificity for α2,6-SAs, allowing the virus to still transmit via aerosol. This mutation occurs naturally and has been documented in cases of severe pdm2009 infection [106,107,108]. However, the mutation did not affect the antigenic properties of the virus, suggesting that the vaccine for pdm2009 would still be effective against viruses with this mutation [109]. The mutation E391K in the HA protein of pdm2009 has been globally increasing in prevalence. Maurer-Stroh et al. sought to find a role this mutation might play and why it may be under selective pressure. E391K has mainly been seen in clinical cases with mild symptoms. While the authors were able to map the mutation to a location that could potentially alter membrane fusion and antigenicity, more studies are needed to determine if a mutation at this residue could alter the biology or fitness of the pdm2009 H1N1 virus [110]. Zhang et al. noted that two glycosylation sites (142N and 177N) on the receptor binding domain of HA are present in most pre-2009 seasonal H1N1 isolates but are absent in all pdm2009 strains. The authors found that adding these glycosylation sites to the pdm2009 HA increased virulence and pathogenicity in mice compared to the wild-type pdm2009 virus. The addition of these glycosylation sites can also reduce sensitivity to neutralizing antibodies from the wild-type pdm2009 virus [111,112].

#### 2.4.2. Mutations Involved in Pathogenicity, Virulence, Replication, and Zoonotic Transmission

Matrosovich et al. found that E190D and G225E mutations in the HA protein in swine H1 viruses increased HA affinity for α2,6-SAs. The authors noted that amino acid substitutions at these residues are also found in human H1 viruses, including 1918 isolates. They suggest that these residues are important for the generation of human H1 pandemic strains and that since these positions mutate rapidly upon introduction from an avian host to a mammal, the mutations may be prerequisites for efficient replication and transmission in mammals [11]. Zhang et al. found that by mutating position 225 from aspartic acid to glycine in the HA of pdm2009 or the HA of the 1918 pandemic virus, the receptor binding specificity changed from α2,6-SA linkage to dual receptor binding. This mutation occurred naturally in the later stages of the 2009 H1N1 pandemic. They propose that the glycine at position 225 disrupts the salt bridge between the aspartic acid originally at position 225 and the lysine at position 222. This loss enables residue Q226 to interact with α2,3-SA linkages [112]. The dual receptor binding was also found to increase replication and transmissibility in the ferret model [113].

### 2.5. The NP Gene Segment

The NP protein plays a critical role during viral RNA synthesis by triggering the progression from transcription to replication mode and has also been implicated in contributing to host specificity. Gabriel et al. demonstrated that the adaptation of PB2 and NP to importin α1 plays an important role in interspecies transmission. They showed that the adaptive mutation PB2 D701N, in combination with NP N319K, allowed an avian virus to adapt to a mouse model. These mutations enabled enhanced binding of PB2 and NP to importin α1, which improved the efficiency of their transport into the nucleus of mammalian cells, leading to enhanced viral replication [64,114]. Sakabe et al. found that NP D375N substantially increased the virulence of a pdm2009 isolate compared to other mutations found in the study. NP 375D is conserved in avian, most classical swine viruses, and most pdm2009 H1N1 viruses, while most seasonal viruses possess valine or glycine at this position [4,26,115]. Consequently, the authors believe that this substitution may play a role in host-range alteration, from avian to mammal species in particular [115]. Cellular antiviral Mx1 in mice or MxA in humans can interact with NP and inhibit influenza replication [116,117]. There have been several cases of NP adaptation mutations to circumvent MxA. These four mutations in NP—16D, 283P, 313Y, and 357K—act as specific markers discriminating humans from avian viruses and were likely required for transmission of the 1918 precursor virus to humans [118]. Currently, circulating human influenza A strains still contain these adaptive mutations, perhaps under the selection pressure of MxA [119]. Manz et al. were able to show that the pdm2009 virus developed mutations for MxA resistance that differed from the 1918 mutations, specifically E53D, R100V, and F313V, which increased resistance. However, an additional seven mutated residues were required to achieve a resistance that was comparable to pdm2009 NP [119].

#### 2.5.1. Role in pdm2009

The NP, HA, and NS gene segments of the pdm2009 virus all originated from the 1918 pandemic H1N1 virus [17].

#### 2.5.2. Mutations Involved in Pathogenicity, Virulence, Replication, and Zoonotic Transmission

Ince et al. sought to explore the mutational evolution of a mouse-adapted virus in guinea pigs. They found that the NP gene alone played a significant role in viral adaptation in guinea pigs. In addition to substitutions in the M1 gene, the authors saw three substitutions in the PA gene: A336T, F346S, and T378A. All the mutations identified are clustered in the region thought to influence the NP-PB2 interaction [120,121,122].

#### 2.5.3. Enhancement of the Effects of Other Gene Segments/Co-Mutations

The residue NP 319K, in concert with PA 615R, has been implicated as a potential contributor to higher virulence in mammals, in part due to their presence in a highly pathogenic human H5N1 isolate [76].

### 2.6. The NA Gene Segment

The activity of the NA protein is critical for viral budding. Following the completion of viral budding from the host cell, the HA receptor still binds to the sialic acids on the surface of the host cell. The NA protein cleaves the terminal sialic acids from the host cell glycans, allowing for virion release. The neuraminidase also cleaves sialic acids off the virion glycoproteins to prevent aggregation [103].

#### 2.6.1. Role in pdm2009

Similar to the matrix gene in the pdm2009 virus, the neuraminidase gene also originated from the Eurasian swine virus, which in turn obtained the segment from the avian influenza virus in 1979 [123]. The balanced activity of HA and NA has been shown to be crucial for the pdm2009 H1N1 virus to adapt to humans, and this balance has been seen in human strains but not precursor swine viruses [124,125].

#### 2.6.2. Enhanced Drug Resistance

Lin et al. proposed that the PB1 mutation S216G that appeared following the 2009 pandemic and led to a lower-fidelity RdRp induced the development of additional mutations in the NA protein associated with oseltamivir resistance. Before 2009, NA H275Y was the exclusive resistance gene; however, following 2009, four additional mutations (S246N, D198G, D198N, and Y155H) were identified and are associated with drug resistance [44].

### 2.7. The M Gene Segment

The matrix gene encodes for the M1 protein and the M2 ion channel. During viral entry, hydrogen ions are pumped into the virion via the M2 ion channel, lowering the pH, causing disruption of protein interactions, and enabling the subsequent release of viral RNPs into the host cell cytoplasm. M2 is also a target of the anti-viral drug amantadine. M1 interacts with vRNA and NP in the host cell nucleus, allowing for interaction in the RNP complex; it also interacts with the nuclear export protein (NEP), leading to the nuclear export of vRNA segments. The M1 protein is also thought to play a role in gathering together viral components for final packaging at the host cell membrane [103].

#### 2.7.1. Role in pdm2009

The matrix gene of the pdm2009 virus originated from an Eurasian swine influenza virus, which acquired the segment from an avian influenza virus in 1979 [126]. It has been demonstrated that the matrix gene was critical for the highly transmissible phenotype that was seen in pdmH1N1. Chou et al. verified this in the guinea pig model, demonstrating that the matrix gene segment alone from the pandemic strain A/California/4/2009 (H1N1) was able to rescue the transmissibility of the previously non-transmissible A/Puerto Rico/8/1934 (H1N1) [127]. These findings were recapitulated in transmission studies in swine [128] and ferrets [129], confirming the importance of the matrix gene in viral transmission. These findings are further supported by the regular spillover events of variant swine H3N2 viruses into humans. All of the H3N2 viruses infecting humans contain the matrix gene from the 2009 pdmH1N1, supporting its role in transmission to humans [130].

Recent data from Calderon et al. found that the limited replicative capacity of avian influenza viruses in mammalian hosts could be partly caused by dysregulation of gene expression in the matrix gene segment. They demonstrated that when the matrix gene segment of an avian virus is transcribed within a mammalian cell, the segments create more M2 protein than M1. When expressed at high levels, M2 can disrupt vesicular homeostasis, which in turn can reduce viral growth. This regulation of viral gene expression is a novel host-dependent feature of the influenza life cycle, contributing to the host range restriction of a virus [131]. The authors observed that adaptations of reduced levels of M2 production of avian viruses arose previously, allowing avian viruses to replicate in a mammalian host. One such event can be seen with the Eurasian avian-like swine lineage matrix gene that emerged from birds in the late 1970s and contributed the matrix gene segment to the pdm2009 virus.

#### 2.7.2. Mutations Involved in Pathogenicity, Virulence, Replication, and Zoonotic Transmission

Several of the viral proteins in influenza can directly affect replication, transmission, and virulence in the host. Specific mutations at certain sites within the proteins can drastically alter the outcome of the infection. A mutation at residue 86 within the M2 protein can lead to increased viral replication in a temperature-dependent manner [132]. Wohlgemuth et al. mutated residue 86 in the M2 protein from an alanine to a serine on a LAIV backbone. They observed increased replication in differentiated primary human nasal epithelial cell cultures at 37 °C compared to the LAIV containing M2 with alanine at residue 86. However, they did not see this difference at 32 °C. In addition, replacing an alanine with a proline at residue 41 of the M1 protein can result in a reduction in transmission efficiency [133]. Residue 41 of the M1 protein has also been linked to virulence. Replacing alanine at position 41 with valine has been seen to cause increased virulence in a mouse model [134,135]. A temperature-sensitive variant of A/WSN/33, referred to as A/WSN/33 *ts51,* contains an F79S amino acid substitution in the M1 gene. This mutation creates a novel phosphorylation site, which, at a non-permissive temperature, can lead to hyperphosphorylation and accumulation of M1 in the nucleus, inhibiting its function in the cytosol to form infectious virions at the plasma membrane [136]. Liu et al. observed that the double mutant R101S with R105S in M1 on the A/WSN/33 background caused a temperature-sensitive phenotype in vitro and an attenuated phenotype in vivo [137]. Fan et al. demonstrated that the M1 protein contributes to the virulence of H5N1 avian viruses in a mammalian host. They described the importance of an Asp at residue 30 and an Ala at residue 215 in the M1 protein for H5N1 lethality in a mouse model [138]. Ince et al. expanded the findings of Chao et al. on the impact of the M1 gene on transmissibility by demonstrating that either F62L or V166M mutations in the M1 gene enhanced replication and transmission in a guinea pig model [121]. Additional residues in the M1 gene have been found to be linked with viral morphology. Bialas et al. noted that pdmH1N1 virions were predominantly spherical in morphology versus North American triple reassortment swine viruses, which were more filamentous. By comparing differing residues in the M1 gene, they found that positions 30S, 207N, and 209T on the M1 gene are involved in viral morphology and viral transmission in vitro [139].

#### 2.7.3. Enhancement of the Effects of Other Gene Segments/Co-Mutations

Miotto et al. conducted a large-scale complete-proteome analysis of 23,000 influenza A virus sequences across avian, human, swine, and equine species. They identified 68 characteristic sites that are highly conserved in human influenza isolates but rarely observed in avian isolates. They are likely to be associated with important adaptive mutations that allow the virus to propagate in human hosts. They found that the M2 protein contained the highest density of characteristic sites, about 1 for every 10 residues, indicating the importance of matrix gene mutations in host adaptation and possible transmission to and between humans. Specifically, they identified characteristic sites in the reciprocal binding regions of both M1 and NEP/NS2, proposing that these mutations possibly co-evolved due to preferred structural interactions [140]. The pdmH1N1 shares many characteristic sites with currently circulating swine viruses, lending to the possibility that low-pathogenicity human infections by these strains occur. It is possible that developed immunity to pdmH1N1 will protect against widespread infection with potentially pandemic swine influenza. However, as demonstrated by the emergence of pdmH1N1, a reassortment of gene segments between human, avian, and swine isolates can result in a novel virus for which limited or no immunity is present. Other gene constellations may improve fitness and transmission. For example, prior studies have shown that the pandemic origin matrix gene increases NA activity and transmissibility, suggesting a fitness benefit [129,133,141,142].

#### 2.7.4. Enhanced Drug Resistance

The matrix gene is also responsible for enhanced drug resistance. The presence of mutations in the M2 protein has led to adamantane resistance, in particular S31N and V27A, as well as the less common mutations L26F and A30T [143]. Using a computational 3D structural rendering of M2, Du et al. were able to discover a mutation, L43T, in the M2 protein of the 2009 H1N1 virus that may have a negative impact on the bioactivity of rimantadine. This is due to the replacement of a non-polar residue with a polar residue located very close to the functional residue, Trp44, which is the channel lock and binding site of rimantadine [144].

**Table 1:** Fitness determinants of influenza A viruses.

Segment	Amino Acid Position	Subtypes	Remarks	References
PB2	E627K	H1N1, H3N2, H5N1	Did not lead to enhanced virulence or transmission in pdm2009 H1N1, replicates more efficiently in mammalian cells before 2009, increased lethality in mice before 2009, increased H5N1 virulence in mice, increased H5N1 transmissibility in ferrets, determinant of cold sensitivity	[45,46,52,54,55,57,58,59,72]
	T271A, 590S, 591R	H1N1	Critical for viral replication and virulence of swine viruses in vitro and in vivo, associated with mammalian adaptation	[47,48,61]
	E667G	H1N1, H5N1	Associated with mammalian adaptation, did not lead to enhanced virulence or transmission	[45,49]
	T588I	H1N1	Increased polymerase activity and viral replication in mammalian cells, increased pathogenicity in mice, and regulated host antiviral innate immune responses in vitro and in vivo	[51]
	D701N	H1N1, H3N2, H5N1	Did not lead to enhanced virulence or transmission in pdm2009 H1N, associated with mammalian adaptation, improved viral growth in mammalian cells, and enhanced aerosol transmission in guinea pigs of H3N2 and H5N1	[45,46,61,62,63,64]
	526R	H3N2, H5N1, H7N9	Increased polymerase activity, in concert with 627K, enhanced replication and virulence in mice and was associated with mammalian adaptation	[65]
	E158G	H1N1	Increased virulence in mice and increased polymerase activity in human cells	[66]
	I147T, K339T, A558T	H1N1, H5N1	Increased replication in mammalian cells and enhanced virulence in mice	[67,68]
	V661A, A683T/A684S	H1N1	Enhanced viral replication at 34 °C	[50]
PB1	V336I	H1N1	Associated with mammalian adaptation	[71]
	S216G	H1N1	Associated with mammalian adaptation, attenuated virulence in mice, and reduced RdRp fidelity	[44,71]
	473V	H5N1, H1N1	Maintaining efficient viral replication	[72]
	L13P; S678N	H1N1	Enhanced polymerase activity	[75]
	G622D	H5N1	Decreased polymerase activity and attenuation in mice	[69]
	K577E	H9N2	Increased pathogenicity in mice and was associated with mammalian adaptation	[77]
	M317I	H5N1	Increased pathogenicity in mice	[78]
	N375S	H1N1, H2N2, H3N2	Associated with mammalian adaptation	[79]
	K/I340; K/I649, T667	H1N1	Increased virulence in mice	[75,80]
	A469T	H1N1	Enhanced polymerase activity, transmissibility in guinea pigs, and potential pathogenicity determinant	[81]
	N66S	H1N1, H5N1	Increased pathogenicity in mice	[84]
PA	T85I, G186S, L336M	H1N1	Enhanced polymerase activity and enhanced morbidity in mice	[95]
	V100I	H1N1	Regulation of translation and accumulation of viral mRNA (in combination with T85I and G186S)	[96]
	A343T, K353R, T566A	H1N1	Enhanced replication and virulence in mice	[97]
	K356R	H1N1	Associated with mammalian adaptation	[71]
	T97I	H5N2	Associated with mammalian adaptation	[98]
	V44I, V127A, C241Y, A343T, I573V	H5N1	Increased replication in mammalian cells and enhanced virulence in mice were associated with mammalian adaptation	[99]
	K142Q	H5N1	Enhanced replication and pathogenesis in mice when combined with PB2 627K, associated with mammalian adaptation	[101]
	V100I, N321K, I330V, A639T	H1N1	Increased replication and pathogenesis in mice and increased disease severity and transmission in ferrets	[100]
	L295P	H1N1	Increased polymerase activity in mice when combined with PB2 E158G/A	[102]
HA	E391K	H1N1	Currently, in pdm2009, viruses circulating potentially alter membrane fusion and antigenicity	[110]
	142N, 177N	H1N1	Increased virulence and pathogenicity in mice, reduced sensitivity to neutralizing antibodies	[111,112]
	E190D, G225E	H1N1	Increased receptor binding affinity, associated with mammalian adaptation	[11]
	D225G	H1N1	Altered receptor binding specificity, increased replication, and transmissibility in ferrets	[106,107,108,109,112,113]
NP	N319K	H7N7	Associated with mammalian adaptation when combined with PB2 D701N	[64,114]
	D375N	H1N1	Increased virulence in mice, associated with mammalian adaptation	[4,26,115]
	16D, 283P, 313Y, 357K	H1N1	Circumvent antiviral MxA	[118,119]
	E53D, R100V, F313V	H1N1	Increased resistance to MxA	[119]
	A336T, F346S, T378A	H1N1	Adaptation to guinea pigs	[120,121,122]
	319K	H5N1	Greater virulence in mammals when combined with PA 615R	[76]
	N319K	H7N7	Associated with mammalian adaptation when combined with PB2 D701N	[64,114]
NA	H275Y, S246N, D198G, D198N, Y155H	H1N1	Oseltamivir resistance	[44]
M	A86S	LAIV—H2N2 backbone, H3N2	Increased viral replication in a temperature-dependent manner	[132]
	A41P	H1N1	Reduction in transmission efficiency	[133]
	A41V	H1N1	Increased virulence in mice	[134,135]
	F79S	H1N1	Attenuated replication	[136]
	R101S, R105S	H1N1	Temperature-sensitive in vitro, attenuation in vivo	[137]
	30D, 215A	H5N1	Increased virulence in mice	[138]
	30S, 207N, 209T	H1N1	Viral morphology	[139]
	F62L; V166M	H1N1	Enhanced replication and transmission in guinea pigs	[121,127]
	S31N, V27A, L26F, A30T	H1N1, H3N2	Adamantane resistance	[143]
	L43T	H1N1	Rimantadine resistance	[144]

### 2.8. The NS Gene Segment

NS1 is a non-structural protein that is a non-essential virulence factor. Its major role has been described as an inhibitor of host immune responses, particularly the inhibition of interferon (IFN) production and the antiviral effects triggered by the production of IFN. The NS1 also modulates viral RNA replication, viral protein synthesis, and other important aspects of the viral replication cycle [145]. Seo et al. observed that lethal H5N1 viruses (specifically human H5N1 isolates from 1997) are resistant to the antiviral effects of IFNs as compared to human, swine, and other avian influenza viruses. The authors found that a recombinant human H1N1 containing the NS gene segment from a lethal H5N1 1997 virus was more virulent and pathogenic in swine than the parental virus. They found that this effect was dependent on glutamic acid at position 92 in the NS1 protein [146]. The NS1 protein can interact with a cellular factor, CPSF30, to block the processing of cellular mRNAs, consequently leading to the general inhibition of cellular gene expression, including antiviral genes and proinflammatory cytokine genes [147,148,149,150,151]. This binding is not conserved across all influenza A subtypes.

#### 2.8.1. Role in pdm2009

The NS1 protein of pdm2009 viruses is truncated at position 220; this eliminates a PDZ ligand binding domain. The PDZ ligand domain is located on the C-terminus of the NS1 protein and can be used to bind proteins that aid in the transport, localization, and assembly of signaling complexes. The PDZ ligand domain has been proposed as a virulence determinant. These four C-terminal residues are found on H5N1 viruses as well as the 1918 virus strains [152].

Kanrai et al. found three mutation residues in the NS gene that can contribute to the adaptation of a virus from an avian to a mammalian host. They found that substitution of 3S, 41K, and 74N enabled increased viral titers of avian viruses in human cells. However, they also noted that these substitutions did not affect the viral growth in avian cells. They were also able to show that a pdm2009 H1N1 isolate already containing the 3S and 41K residues demonstrated enhanced replication in mammalian cells with the addition of residue 74N. The mechanism behind these mutations is currently unknown [153]. The pdm2009 H1N1 viruses are unable to suppress host innate immune responses via gene expression inhibition due to inefficient binding of cellular CPSF30 but are still effectively antagonizing IFN production. Hale et al. introduced three mutations into the NS gene segment of a pdm2009 isolate. These mutations changed residues that are highly conserved in the NS1 proteins of classical swine viruses to residues that correspond to a human virus consensus sequence. They found that the introduction of R108K, E125D, and G189D enabled a pdm2009 isolate to antagonize the host innate immune response more efficiently in primary human epithelial cells without impacting viral replication. This mutant virus also showed less morbidity in a mouse model and less viral replication in a ferret model [154]. Initially, pdm2009 viruses were not able to bind CPSF30 and inhibit host gene expression. However, Clark et al. discovered pdm2009 viruses circulating during the 2015-16 season that possessed six mutations, allowing the NS1 protein to inhibit host gene expression. They found that NS1 E55K, L90I, I123V, E125D, K131E, and N205S were selected for inclusion in the population, and now almost all circulating pdm2009 strains contain these mutations [147].

#### 2.8.2. Mutations Involved in Pathogenicity, Virulence, Replication, and Zoonotic Transmission

Dankar et al. found that the mutations F103L and M106I in the NS1 protein increased viral replication, IFN antagonism, and virulence in a mouse model [155]. Manz et al. examined the protein products of human H5N1 viruses to determine how they were able to cross the host barrier. As mentioned previously, they found the PB2-E627K mutation to play an important role. However, they found that viruses not containing this mutation possessed an M16I mutation in the NEP protein. The authors propose that the mutation in the NEP protein enables greater replication of the genome in humans. They also mention several other mutations that have been found in the NEP protein (Y41C, E75G, and S7L), furthering their claim that NEP is a pathogenicity factor important for the host adaptation of avian viruses to a mammalian host [156]. Wei et al. found two mutations in the NS gene segment, among other mutations, after nine serial passages of a pdm2009 virus in pigs. Using reverse genetics, they demonstrated that the NS1 N205K and NEP T48N mutations combined enhanced viral replication in guinea pigs and conferred enhanced contact transmissibility of the virus [81]. Jiao et al. found that the mutation P42S in an H5N1 isolate enhanced the virulence of the avian virus in mice. They showed that this mutation contributed to the antagonization of the cellular antiviral immune response [157].

## 3. Conclusions

Due to its extensive host range allowing for an antigenic shift and the error-prone polymerase enabling antigenic drift mutations, IAV has the potential to cross species barriers and rapidly escape existing immunity, enabling the emergence of novel, potentially pandemic subtypes and viruses. Thus, mutations acquired through both drift and shift allow for changes in host tropism, transmission, and pathogenesis. There are a multitude of mutation sites across the IAV genome where amino acid changes can have a measurable effect and, in some cases, lead to co-mutations, amplifying the phenotype.

Following the 2009 influenza pandemic, pdmH1N1 replaced the previously circulating H1N1 virus and now circulates as a seasonal influenza virus. Repeated introductions of human seasonal influenza viruses into swine have led to the incorporation of human influenza gene segments into circulating swine influenza viruses. This was also seen with the pdmH1N1 virus. While the full pdmH1N1 virus does not circulate in the swine population, certain gene segments have been maintained and, in some cases, have become dominant in current circulating swine viruses, suggesting a fitness benefit. The 2009 H1N1 pandemic was not the most lethal influenza pandemic. However, studies of the pdmH1N1 gene segments demonstrate that components of this virus can lead to increased disease in humans and animal models. Many of these segments or mutations are found in circulating swine influenza viruses.

The regular emergence of pandemic influenza viruses demonstrates the critical need for surveillance and risk assessment of zoonotic influenza viruses. Understanding genetic determinants that affect fitness, virulence, transmission, and host tropism, as well as the mutations that alter these features, provides a reference for potential pandemic genetic screening tools. Knowledge of these mutation sites and attenuating residues may also be used in the development of attenuated influenza virus vaccines and the formulation of new anti-viral drugs. Influenza virus genetic mutation and evolution are inevitable. Through the utilization of surveillance for known genetic determinants as well as continuing research defining novel mutations, we can continue to be prepared for novel, potentially pandemic influenza viruses. Further, using these findings to develop new vaccines and treatments will better prepare us for the next influenza pandemic.
